# What’s the Matter with MICs: Bacterial Nutrition, Limiting Resources, and Antibiotic Pharmacodynamics

**DOI:** 10.1128/spectrum.04091-22

**Published:** 2023-05-03

**Authors:** Brandon A. Berryhill, Teresa Gil-Gil, Joshua A. Manuel, Andrew P. Smith, Ellie Margollis, Fernando Baquero, Bruce R. Levin

**Affiliations:** a Department of Biology, Emory University, Atlanta, Georgia, USA; b Program in Microbiology and Molecular Genetics, Graduate Division of Biological and Biomedical Sciences, Laney Graduate School, Emory University, Atlanta, Georgia, USA; c Centro Nacional de Biotecnología, Consejo Superior de Investigaciones Científicas (CSIC), Madrid, Spain; d Programa de Doctorado en Biociencias Moleculares, Universidad Autónoma de Madrid, Madrid, Spain; e Department of Infectious Diseases, St. Jude Children's Research Hospital, Memphis, Tennessee, USA; f Hospital Universitario Ramón y Cajal, Instituto Ramón y Cajal de Investigación Sanitaria, and Centro de Investigación Médica en Red, Epidemiología y Salud Pública (CIBERESP), Madrid, Spain; Hartford Hospital

**Keywords:** antibiotics, minimum inhibitory concentration, pharmacodynamics, population biology, antibiotic resistance, heteroresistance

## Abstract

The MIC of an antibiotic required to prevent replication is used both as a measure of the susceptibility/resistance of bacteria to that drug and as the single pharmacodynamic parameter for the rational design of antibiotic treatment regimes. MICs are experimentally estimated *in vitro* under conditions optimal for the action of the antibiotic. However, bacteria rarely grow in these optimal conditions. Using a mathematical model of the pharmacodynamics of antibiotics, we make predictions about the nutrient dependency of bacterial growth in the presence of antibiotics. We test these predictions with experiments in broth and a glucose-limited minimal media with Escherichia coli and eight different antibiotics. Our experiments question the sufficiency of using MICs and simple pharmacodynamic functions as measures of the pharmacodynamics of antibiotics under the nutritional conditions of infected tissues. To an extent that varies among drugs: (i) the estimated MICs obtained in rich media are greater than those estimated in minimal media; (ii) exposure to these drugs increases the time before logarithmic growth starts, their lag; and (iii) the stationary-phase density of E. coli populations declines with greater sub-MIC antibiotic concentrations. We postulate a mechanism to account for the relationship between sub-MICs of antibiotics and these growth parameters. This study is limited to a single bacterial strain and two types of culture media with different nutritive content. These limitations aside, the results of our study clearly question the use of MIC as the unique pharmacodynamic parameter to develop therapeutically oriented protocols.

**IMPORTANCE** For studies of antibiotics and how they work, the most-often used measurement of drug efficacy is the MIC. The MIC is the concentration of an antibiotic needed to inhibit bacterial growth. This parameter is critical to the design and implementation of antibiotic therapy. We provide evidence that the use of MIC as the sole measurement for antibiotic efficacy ignores important aspects of bacterial growth dynamics. Before now, there has not been a nexus between bacteria, the conditions in which they grow, and the MIC. Most importantly, few studies have considered sub-MICs of antibiotics, despite their clinical importance. Here, we explore these concentrations in-depth, and we demonstrate MIC to be an incomplete measure of how an infection will interact with a specific antibiotic. Understanding the critiques of MIC is the first of many steps needed to improve infectious disease treatment.

## INTRODUCTION

Fundamental to the rational, as opposed to purely empirical design of antibiotic treatment regimens are the bacterial-dependent pharmacodynamics (PD) (1–3), and host-dependent pharmacokinetics (PK) (1, 2, 4), and their relationship known as PK/PD indices. The PKs of antibiotics are measured from the changes in concentrations of the drug in the serum of treated hosts, often utilizing uninfected volunteers (5, 6). Although different measures of PK of the antibiotic are employed for PK/PD indices (7), most commonly a single measure of the PD of the antibiotic and its target bacteria are employed. This measure is the minimum concentration of the antibiotic necessary to prevent the replication of the bacteria, the MIC (8).

MICs are measured *in vitro*, most commonly by two-times serial dilution of the antibiotic (9, 10). In this method, bacteria are inoculated into a rich media at a density of 5 × 10^5^ cells per milliliter and the antibiotic is added at a high concentration to a single culture. The cultures are serially diluted by a factor of two and incubated for a defined period of time. At this time, the optical densities of the antibiotic-exposed cultures are estimated by eye, looking for the well where turbidity drastically decreases (11). The antibiotic concentration at which this sharp decrease in turbidity is seen is called the MIC. This method tends to have a lower throughput than what is needed for clinical laboratories; thus, other standardized, machine-based protocols are used for estimating MICs. Though, these methods such as the Vitek (12, 13) are also based on optical density, and work by comparing the growth of a known isolate to an unknown isolate. Nonoptical density-based methods do exist, such as Etests (14, 15).

It has been shown that the value of the MIC estimated for antibiotic and bacteria pairs are critically dependent on the density of bacteria exposed to the antibiotics when starting MIC estimation experiments (16). In this investigation, we use mathematical models to explore the relationship between the richness of the media and concentration of a limiting resource, on the pharmacodynamics of antibiotics and bacteria. Using Escherichia coli growing in a highly buffered glucose-limited minimal medium and lysogeny broth (LB), we explore the fit of these models to the pharmacodynamics of antibiotics of eight different classes.

The results of our study show how different factors affect the MIC estimation as determined by standard procedures and question the sufficiency and validity of this pivotal parameter to provide a quantitatively accurate picture of the pharmacodynamics of antibiotics and bacteria (17). We demonstrate that: (i) the PD of antibiotics are critically dependent on the media used; (ii) exposure to sub-MICs of antibiotics increases the time before bacteria start to grow; (iii) exposure to sub-MICs of antibiotics decreases the growth rate of these bacteria in proportion to the concentration of the drug; (iv) the stationary-phase density to which the bacteria grow is inversely related to the concentration of antibiotics; and (v) sub-MICs of antibiotics increase the nutrient requirements of the treated bacteria. We discuss the implications of these PD results to the design and evaluation of antibiotic treatment protocols.

## RESULTS

### A generalized model of the pharmacodynamics of antibiotics and bacteria and its predictions.

For our model of the pharmacodynamics of antibiotics and bacteria, we combine Hill functions (16) with a resource-limited growth model (18). In accord with this extended model, the hourly rate of growth of the bacteria is defined with respect to the concentration of the antibiotic (A; μg/mL) and the limiting resource (r; μg/mL) as shown in equation 1.

(1) $$\prod \left(A,r\right)=v_{\max}-\left[\frac{\left(v_{\max}-v_{\min}\right)\cdot {\left(\frac{A}{\mathrm{MIC}}\right)}^{K}}{{\left(\frac{A}{\mathrm{MIC}}\right)}^{K}-\left(\frac{v_{\min}}{v_{\max}}\right)}\right]\cdot \text{ψ}\left(r\right)$$

The parameter *v_MAX_* is the maximum rate of growth per cell per hour of the bacteria in the absence of the antibiotic, where *v_MAX_* > 0. *v_MIN_* is the minimum growth rate per cell per hour of the bacteria in the presence of the antibiotic, *v_MIN_* < 0. *MIC* is the MIC in μg/mL of the antibiotic. K is a shape parameter that determines the acuteness of the π(*A*, *r*) function, the greater the value of K the more acute. ψ(r), defined in equation 2, expresses the relationship between the rate of growth of the bacteria and the concentration of the limiting resource, *r*. The parameter k in μg/mL is the Monod constant which is the concentration of the resource when the growth rate in the absence of antibiotics is half of its maximum value, *v_MAX_*.

(2) $$\text{ψ}\left(r\right)=\frac{r}{\left(\text{r}+\text{k}\right)}$$

To explore the relationship between antibiotic concentration, limiting resource concentration, and bacterial density, we present three coupled differential equations based on the Hill functions.

(3) $$\frac{\mathrm{dN}}{\mathrm{dt}}=\prod \left(A,r\right)\cdot N$$

(4) $$\frac{\mathrm{dr}}{\mathrm{dt}}=-e\cdot \prod \left(A,r\right)\cdot N$$

(5) $$\frac{\mathrm{dA}}{\mathrm{dt}}=-\mathrm{da}\cdot A$$

∏*(A*, *r)* is the Hill function designated rate of growth or death of the bacteria when the concentration of the antibiotic is A and the resource is r (equation 3). The conversion efficiency, e μg, is the amount of the limiting resource needed to produce a new cell (equation 4). The parameter da is the hourly rate of decline in the concentration of the antibiotic (equation 5).

**FIG 1:**
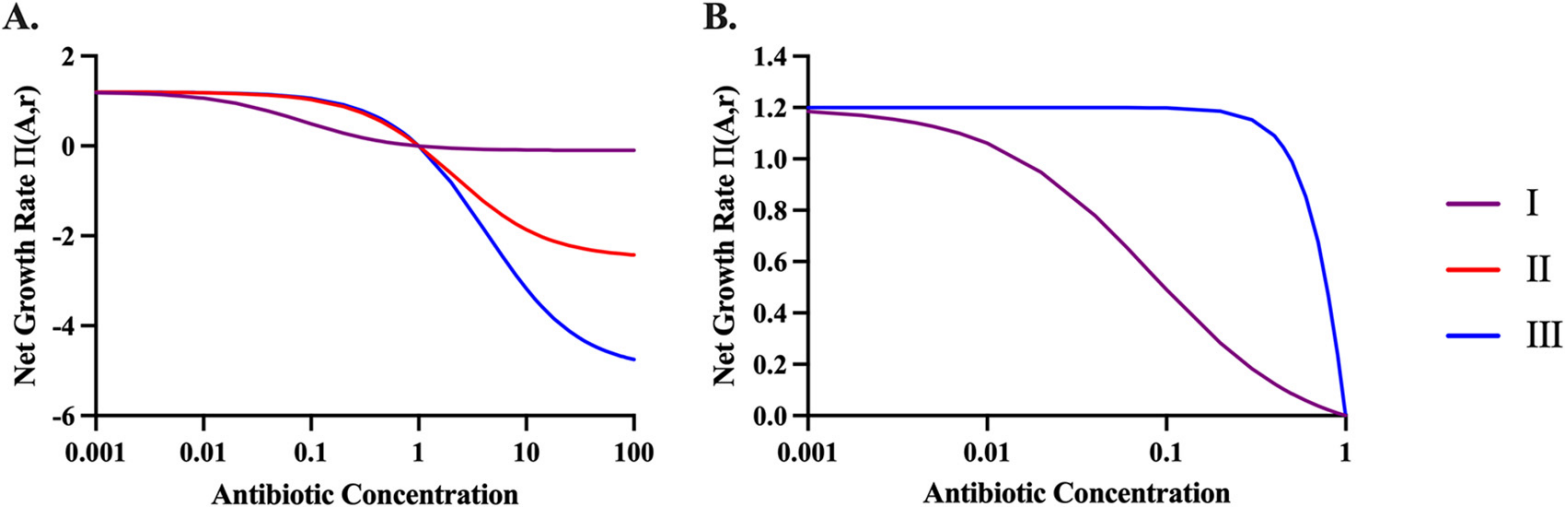
Predicted growth rates of antibiotic-exposed bacteria. To illustrate the properties of this model, we explore three different parameters sets I, II, and III. Condition I represents a bacteriostatic antibiotic (*v_MIN_* = −0.01). Condition II represents a weakly bactericidal antibiotic (*v_MIN_* = −2.5). Condition III represents a strong bactericidal antibiotic (*v_MIN_* = −5.0). Parameters used for these simulations are e = 5 × 10^−7 ^μg/per cell, *v_MAX_* = 1.2 per cell per hour, Κ = 1, k = 1, MIC = 1.0. (A) and (B) The relationship between the concentration of an antibiotic and the rates of growth and death of bacteria π (*A*, *r*) anticipated by the pharmacodynamic function for the described values of the Hill function parameters (equation 1). In these figures the population is not limited by the resource ψ (*r*) = 1.0. (A) All concentrations, (B) Sub-MICs of the antibiotic.

In Fig. 1A and 1B we illustrate the predicted relationship between the concentration of the antibiotic and the rates of growth and death of bacteria in an environment with an indefinite amount of resource, ψ(r) = 1.0, for a bacteriostatic (Condition I), weakly bactericidal (Condition II), and strongly bactericidal (Condition III) antibiotic based on different Hill function parameters. Other than indicating the minimum concentration of the antibiotic that prevents growth, the MIC of an antibiotic provides no information about the dynamics, rate of growth, or rate of death of the bacteria. Antibiotics with the same MIC can be bacteriostatic (Fig. 1A, Condition I) or they can be bactericidal (Fig. 1A, Condition II and Condition III). Moreover, the rate of growth of bacteria exposed to sub-MICs of bacteriostatic antibiotics (Fig. 1B, Condition I) would be less than bacteria exposed to highly bactericidal antibiotics (Fig. 1B, Condition III).

**FIG 2:**
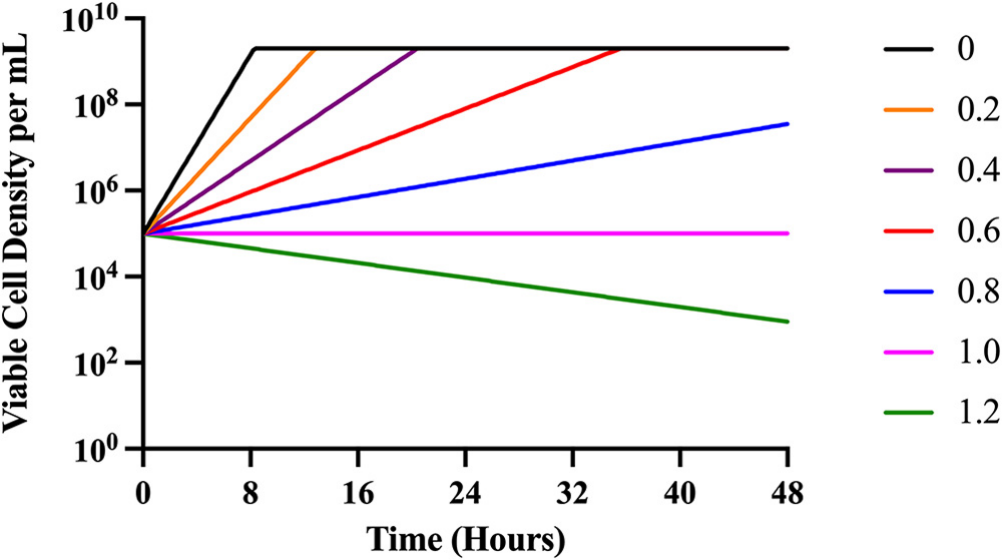
Predicted changes in density of antibiotic-exposed bacteria. To illustrate the properties of this model, we explore only the strong bactericidal antibiotic condition (Condition III). Condition III represents a strong bactericidal antibiotic with *v_MIN_* = 5.0. Parameters used for these simulations are e = 5 × 10^−7 ^μg/per cell, *v_MAX_* = 1.2 per cell per hour, Κ = 1, k = 1, MIC = 1.0. Changes in the viable density of bacteria anticipated from the Hill function model for different concentrations of the antibiotic, respectively, 0 to 1.2 times MIC. In these numerical solutions to equations 2 to 4, we assume the concentration of the limiting resource (r) is 1,000 when the bacteria are first introduced to the antibiotic and the concentration of the antibiotic does not decay, da = 0.

In Fig. 2, we follow theoretical changes in the density of bacteria exposed to different concentrations of a strongly bactericidal antibiotic as predicted by our model (equations 1–5). Under these conditions, the model predicts that the density achieved by the bacterial population is the same at all concentrations of the antibiotic below MIC. Stated another way, the theory predicts that the reduction in growth rate due to the presence of the antibiotic only increases the time before the population reaches stationary phase.

### Experimental exploration of MIC as the primary PD parameter. (i) MICs.

We open our experimental exploration of the pharmacodynamics of antibiotics and bacteria with a consideration of the relationship between the experimentally estimated MICs of antibiotics with *E. coil* MG1655 growing in LB, Davis Minimal (minimal) medium with 1,000 μg/mL glucose, and Mueller-Hinton (MH) (Table 1).

**TABLE 1:** Experimentally estimated MICs of E. coli MG1655 for eight antibiotics in LB, glucose-limited minimal medium, and MH

Antibiotic	LB MIC (μg/mL)	Minimal MIC (μg/mL)	MH MIC (μg/mL)
AZM	25	6.25	6.25
CIP	0.03	0.03	0.03
TET	2.5	0.8	2.5
FOF	37.5	25	37.5
GEN	12	0.75	3
RIF	25	12.5	25
CHL	6.25	6.25	6.25
CRO	0.05	0.03	0.05

For ciprofloxacin (CIP), fosfomycin (FOF), chloramphenicol (CHL), rifampin (RIF), and ceftriaxone (CRO), the MICs in broth and minimal medium are equal or nearly so. For azithromycin (AZM), gentamicin (GEN), and tetracycline (TET), the MICs in broth are substantially greater than that in glucose-limited minimal medium. In the case of MH, all the MICs are the same as in LB with the exception of GEN and AZM which are notably higher in LB. In Table S1, we show the MICs with minimal medium with different carbon sources. Notably, the MICs of certain drugs such as azithromycin, fosfomycin, and ceftriaxone are lower in alternative carbon sources than they are in glucose. The values presented for the MICs of these drugs were chosen as the first concentration at which the optical density declined substantially approached that of the media. As shown in Fig. S1, sub-MICs in most antibiotics lead to a decrease in the optical density in proportion to the drug concentration, an effect we call MIC ooze, that is to say, there is not one clear MIC as the OD decreases stepwise across several wells, rather than precipitously.

**FIG 3:**
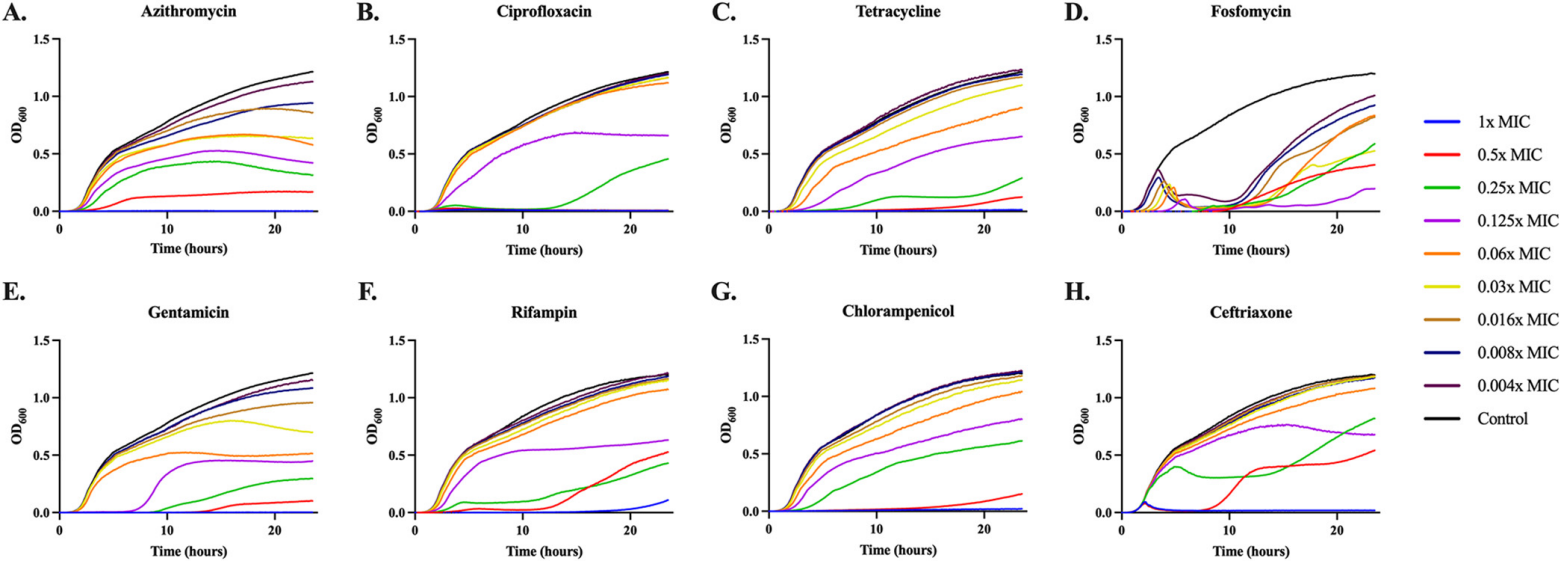
Growth dynamics in LB. Changes in optical density (600 nm) of E. coli MG1655 exposed to different sub-MICs of eight antibiotics for 24 h in LB. Lines are representative of the average of five technical replicas and normalized to the time zero optical density. Each concentration is shown as a fraction of the MIC for the noted drug (Table 1): 1× (blue), 0.5× (red), 0.25× (green), 0.125× (purple), 0.06× (orange), 0.03× (yellow), 0.016× (brown), 0.008× (dark blue), 0.004× (dark purple), and a no drug control (black).

**FIG 4:**
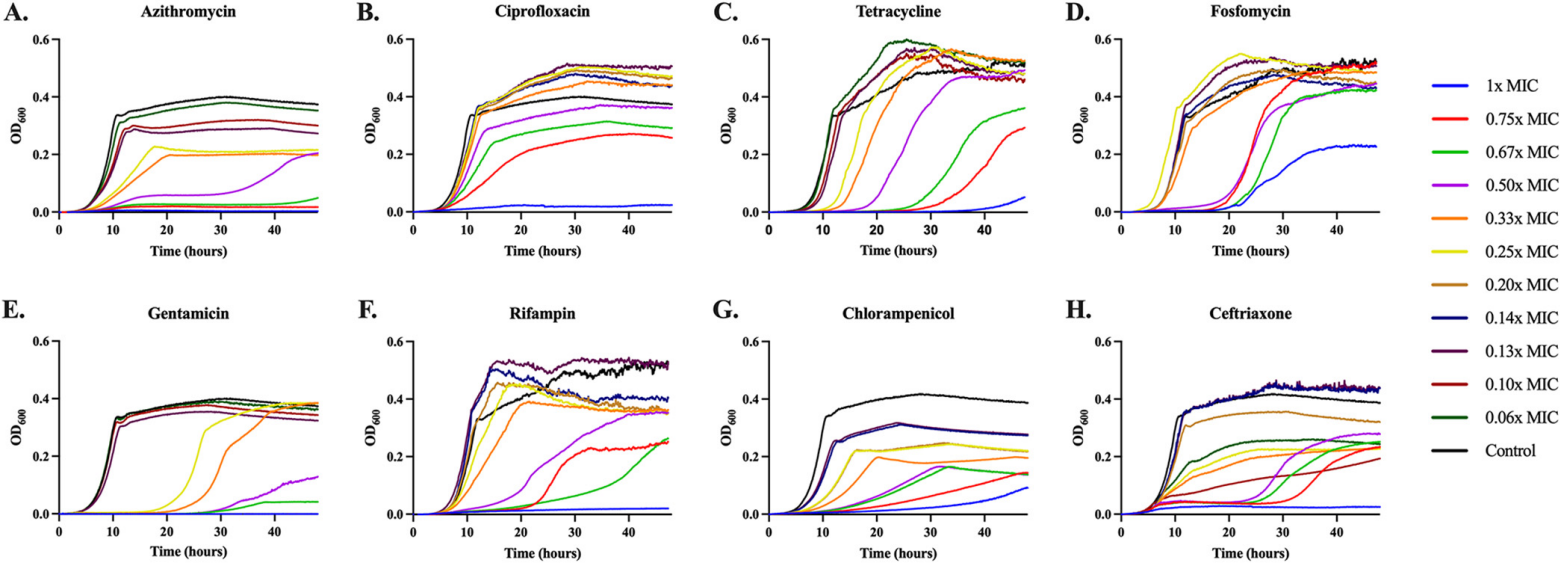
Growth dynamics in minimal medium. Changes in optical density (600 nm) of E. coli MG1655 exposed to different sub-MICs of eight antibiotics for 48 h in glucose-limited minimal medium. Lines are representative of the average of five technical replicas and normalized to the time zero optical density. Each concentration is shown as a fraction of the MIC for the noted drug (Table 1): 1× (blue), 0.75× (red), 0.67× (green), 0.50× (purple), 0.33× (orange), 0.25× (yellow), 0.20× (brown), 0.14× (dark blue), 0.13× (dark purple), 0.10× (dark red), 0.06× (dark green), and a no drug control (black).

**(ii) Growth dynamics at sub-MICs of eight antibiotics in LB and minimal media.** In Fig. 3 and Fig. 4, we follow the changes in optical density of E. coli MG1655 exposed to different antibiotics in LB and minimal media, respectively. A detailed comparison of the parameters estimated from this analysis (maximum growth rate, length of the lag phase, and maximum optical density) are available in Fig. S2 and S5, Fig. S3 and S6, and Fig. S4 and S7, respectively.

The dynamics of change in optical densities of MG1655 vary among the drugs in both broth and minimal medium. For all eight antibiotics, these dynamic data indicate concentration-dependent variation in the time before the populations start to grow, the rate of growth, and the density achieved at 24 h for LB and 48 h for the minimal medium.

In Fig. S2 and S5, we plot the relationship between the estimated maximum growth rates as a function of sub-MIC of the antibiotics in LB (Fig. S2) and minimal (Fig. S5). With the exception of fosfomycin in both media, the max rate of growth increases with the declining concentration of antibiotic. The observation that higher concentrations of the antibiotics would be reducing the growth rate of the bacteria to an extent greater than at lower concentrations is anticipated and central to the use of the pharmacodynamic Hill functions.

In Fig. S3 and S6, we plot the amount of time required before the optical density exceeds 0.020, the relative lag for different concentrations of the antibiotics in LB and minimal medium, respectively. In both media, there is a concentration dependent increase in the lag time, with higher concentrations of the drug leading to longer lag times.

The optical density at 24 h in LB and 48 h in minimal DM are presented in Fig. S4 and S7, respectively. In both media, for all of the antibiotics, the maximum optical density increases with the decline in the concentration of the antibiotic. This decrease in the maximum optical density is associated with a decrease in the viable CFU of these cultures (Fig. S8).

Consistently across all three testing conditions, fosfomycin, gentamicin, and rifampin have high variability between replicates. This high variability can be explained by the emergence of resistance. For fosfomycin and rifampin resistances emerge independently across replicas as shown in Fig. S9 and S10. For fosfomycin an increase in MIC of over 11-fold was seen between pre- and post-antibiotic exposed cultures, while for rifampin an increase of 35 times was seen. This rapid selection for fosfomycin resistance is well known as it has been shown that there is a large inoculum density effect when performing MICs with this drug (19). When MG1655 was exposed to gentamicin, a large number of small colony variants were recovered. One possibility is that the exposure to the drug during LB double dilution rapidly selects for a resistant minority population. As a test of this, we performed an Etest on MG1655 with gentamicin and found the MIC to be 24 times lower by Etest compared to double dilution, a result we believe to be consistent with heteroresistance. All three resistances are observed as a stable increase in MIC.

**FIG 5:**
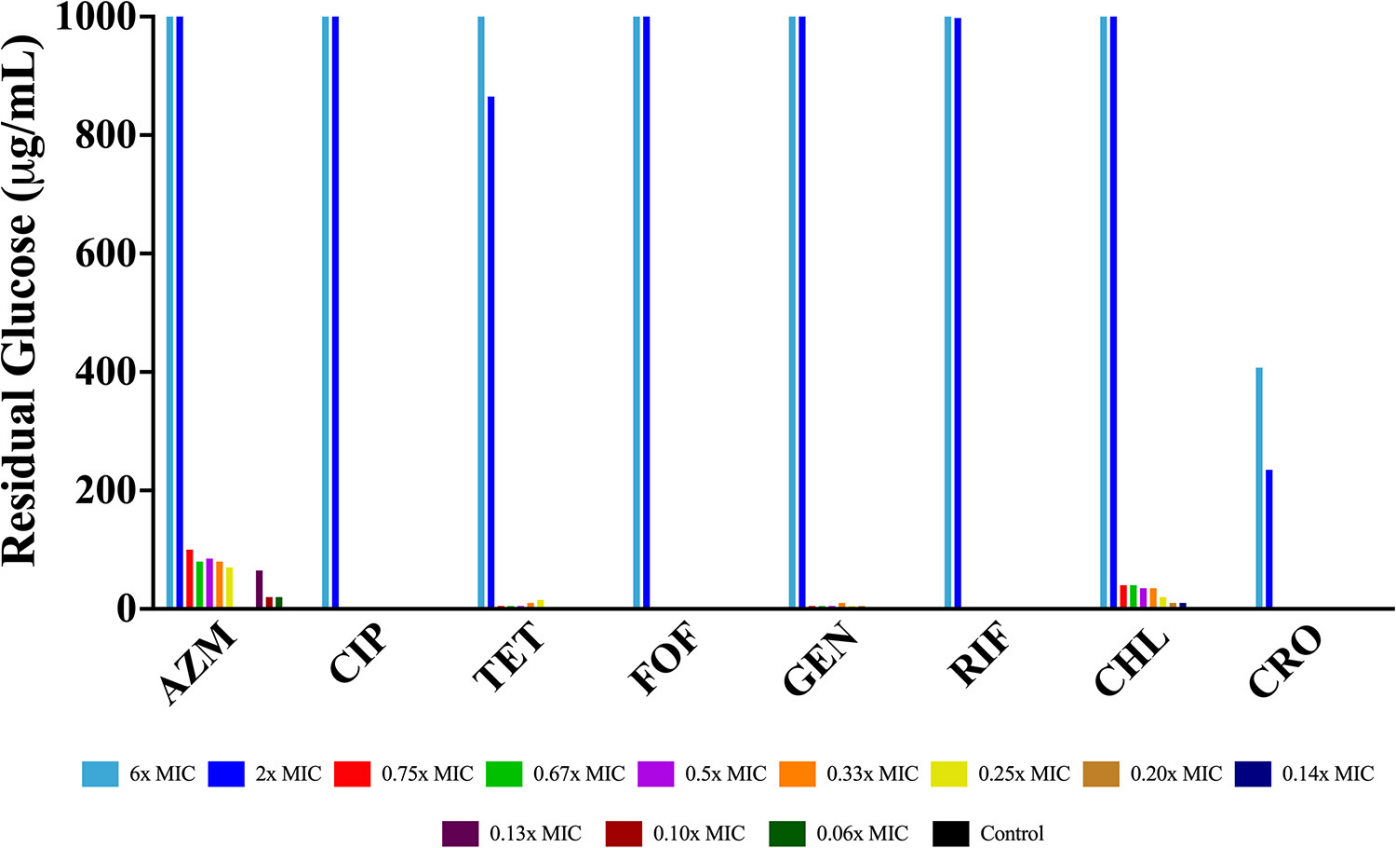
Final glucose concentrations. Amount of glucose (μg/mL) in each culture of E. coli MG1655 exposed to different super- and sub-MICs of eight antibiotics after 48 h of incubation in glucose-limited minimal medium.

**(iii) Consumption of a limited resource in sub-MIC treated cultures.** The association between the concentration of the drug and the final optical density of each culture (Fig. 3 and 4) is striking and inconsistent with the predictions of Fig. 2. In accord with the model, we would expect drug concentration to simply increase the amount of time needed to reach stationary phase, but not to impact the level of stationary phase. This is clearly not the case. One possibility is that when exposed to the drugs, the bacteria become less efficient in the consumption of resource. The utilization of glucose-limited minimal media allows for direct measurement of the glucose level (Fig. 5), and in accord with the above hypothesis, we would expect there to be no excess glucose after 48 h of bacterial growth. Our results are consistent with this hypothesis. At super-MICs of the drugs, we see low to no consumption of the glucose, while at sub-MICs we see consumption of almost all the limiting resource, with the exception of ceftriaxone, which at super-MICs consumes a large amount of glucose.

**FIG 6:**
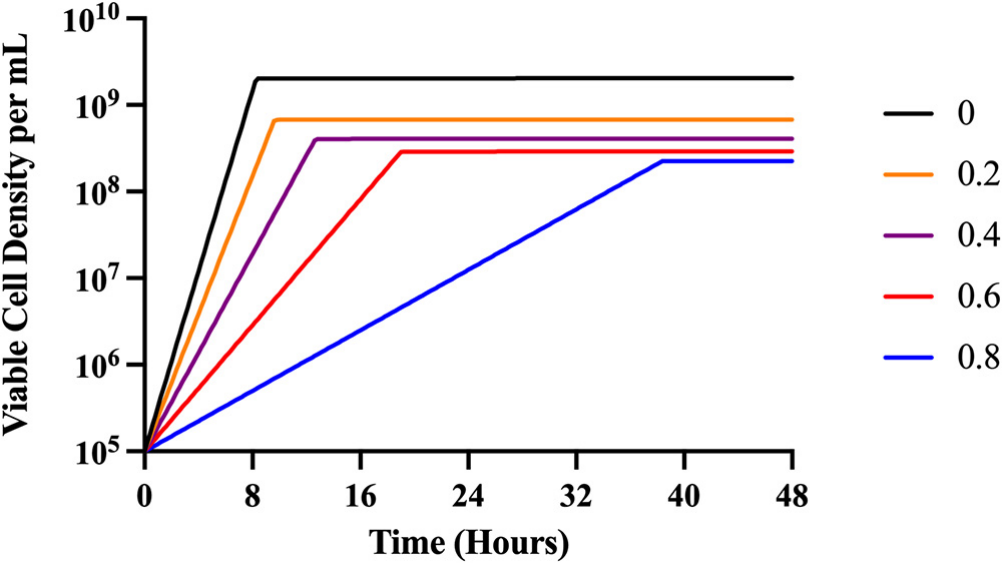
Predicted changes in density of antibiotic-exposed bacteria with antibiotic-dependent resource consumption. To illustrate the properties of this model, we explore only the strong bactericidal antibiotic condition (Condition III). Condition III represents a strong bactericidal antibiotic with *v_MIN_* = 5.0. Parameters used for these simulations are e = 5 × 10^−7 ^μg/per cell, *v_MAX_* = 1.2 per cell per hour, Κ = 1, k = 1, MIC = 1.0. Changes in the viable density of bacteria anticipated from the Hill function model for different concentrations of the antibiotic, respectively, 0 to 0.8 times MIC. In these numerical solutions, we assume the concentration of the limiting resource (*r*) is 1,000 when the bacteria are first introduced to the antibiotic and the concentration of the antibiotic does not decay, da = 0, and that C = 5 × 10^−6^.

**(iv) Antibiotic dependent resource consumption.** In accord with Fig. 2, the stationary-phase densities of bacteria exposed to sub-MICs of antibiotics should be independent of the concentration of the drug. That is clearly not the case as the stationary-phase density declines with the concentration of the antibiotic (Fig. S4 and S7). One possible explanation is that in addition to reducing the rate of growth of the E. coli, the antibiotic increases the amount of resource necessary to produce a new cell (17). As such, we have updated equation 4 to make resource consumption dependent on the concentration of the antibiotic (equation 6). By modifying the resource uptake equation, we get:

(6) $$\frac{\mathrm{dr}}{\mathrm{dxt}}=\left(-e+C\cdot A\right)\cdot \prod \left(A,r\right)\cdot N\;$$

where C is a constant that determines the extent to which e is increased. In Fig. 6, we illustrate the changes in density predicted by this updated model. As shown in Fig. 3 and 4, sub-MICs of antibiotics are able to decrease the stationary-phase density achieved.

**FIG 7:**
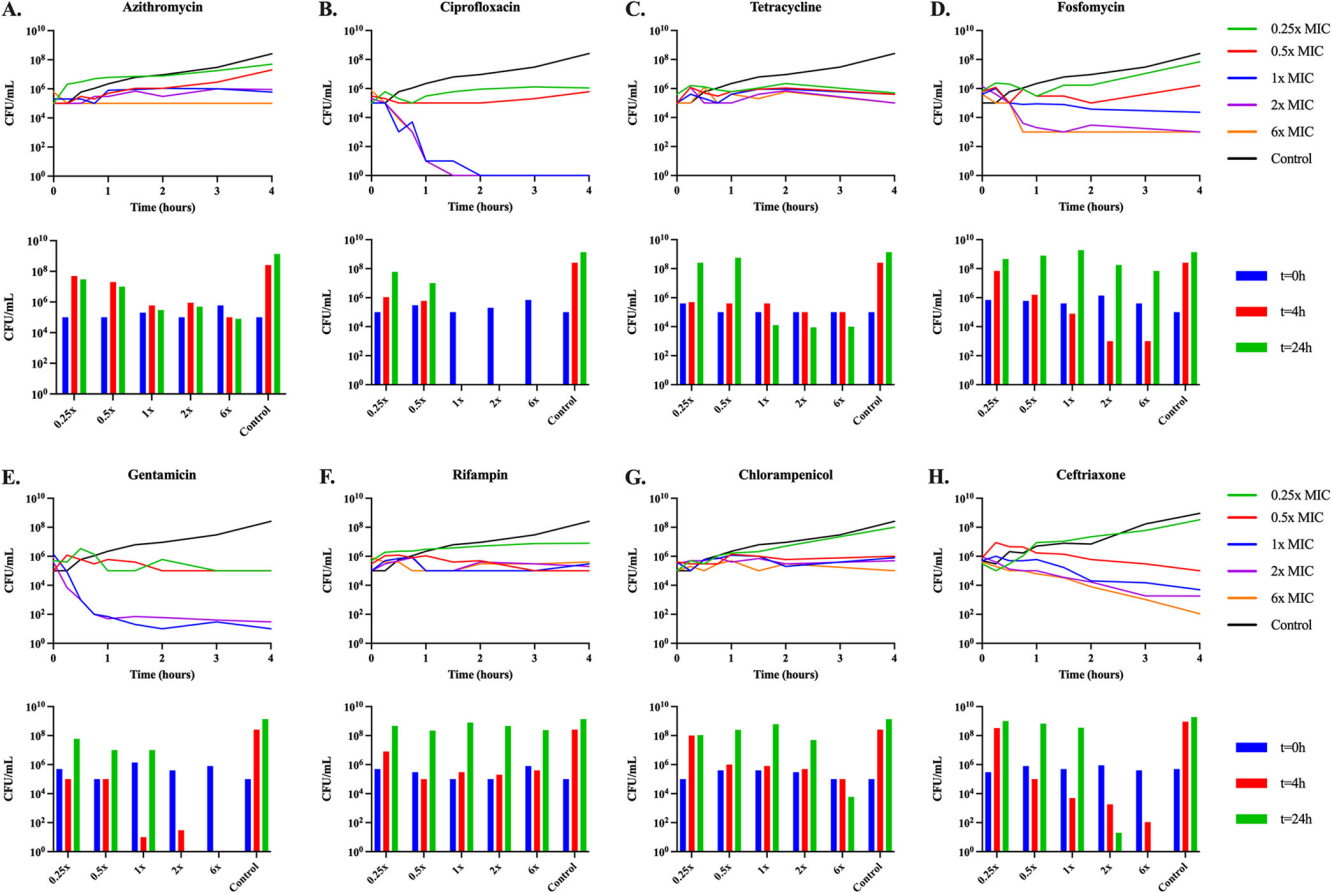
LB time-kills. Changes in viable cell density of E. coli MG1655 exposed to sub- and super-MICs of the eight antibiotics in broth for 24 h. Shown for each panel in the line graph are changes in the viable cell density over a 4-h period, shown in the bar graph is the viable cell density at 0, 4, and 24 h. (A) Azithromycin; (B) Ciprofloxacin; (C) Tetracycline; (D) Fosfomycin; (E) Gentamicin; (F) Rifampin; (G) Chloramphenicol; (H) Ceftriaxone.

**FIG 8:**
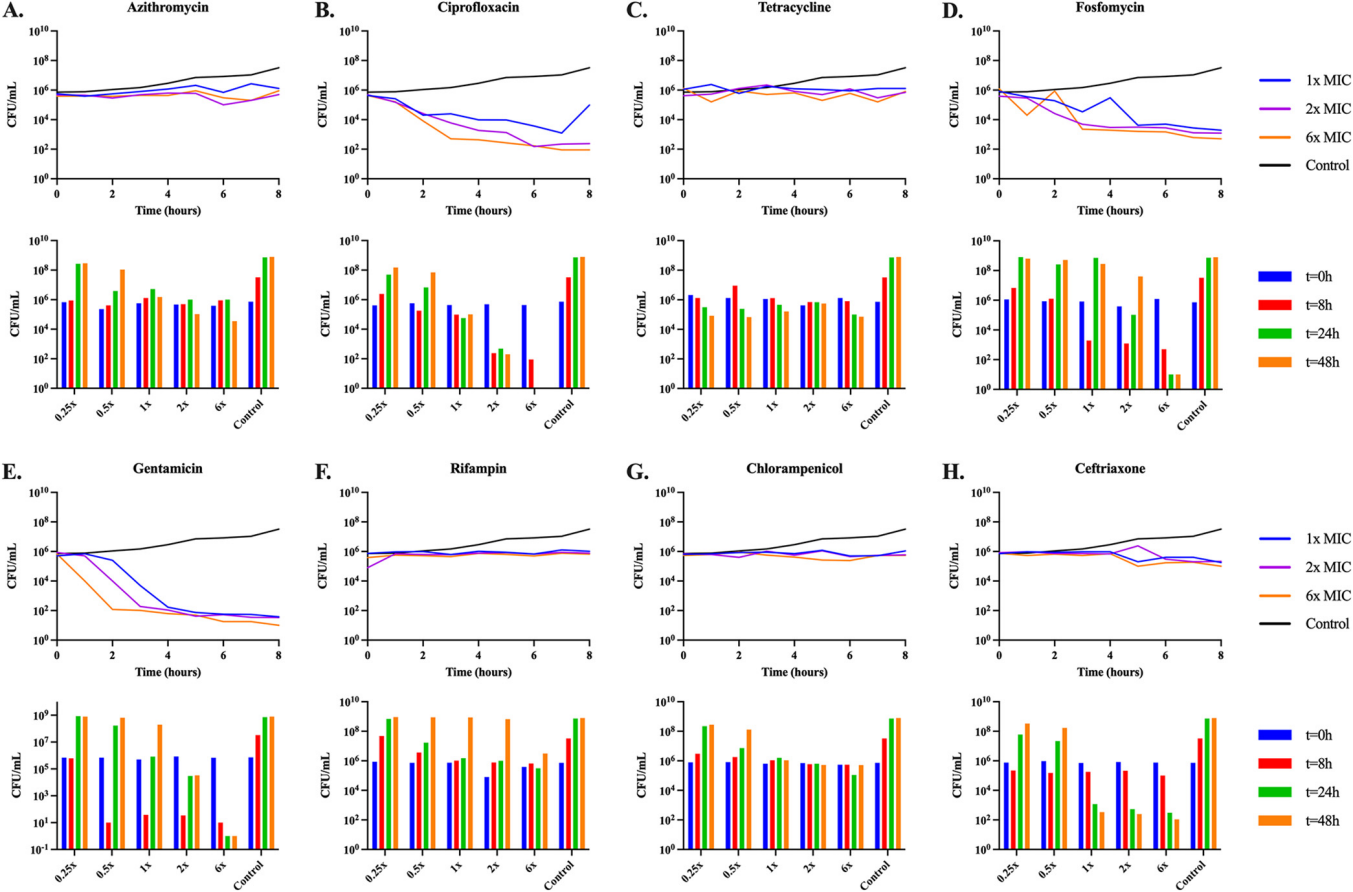
Minimal medium time-kills. Changes in viable cell density of E. coli MG1655 exposed to sub- and super-MICs of the eight antibiotics in minimal for 48 h. Shown for each panel in the line graph are changes in the viable cell density over a 4-h period, shown in the bar graph is the viable cell density at 0, 8, 24, and 48 h. Sub-MICs of the antibiotics are not shown, as over 8 h there was no change in CFU in any of the cultures. (A) Azithromycin; (B) Ciprofloxacin; (C) Tetracycline; (D) Fosfomycin; (E) Gentamicin; (F) Rifampin; (G) Chloramphenicol; (H) Ceftriaxone.

**(v) Rates of antibiotic-dependent growth and mortality.** To explore the relationship between the concentration of the antibiotic and the rates of growth and death of E. coli MG1655, we performed time-kill experiments. Overnight cultures of the bacteria in LB (Fig. 7) or minimal medium (Fig. 8) were mixed with different concentrations of the antibiotics above and below the MIC for the drugs in the noted media. Viable cell densities (CFU) of the cultures were estimated at different times.

In both LB and minimal medium, the bacteriostatic drugs (azithromycin, tetracycline, and chloramphenicol) suppress growth of the bacteria at super-MICs. In both media, ciprofloxacin, gentamicin, and ceftriaxone appear highly bactericidal, although ceftriaxone does not kill at all in the first 8 h in minimal media. Rifampin and fosfomycin are both complicated by the emergence of genotypic resistance. Rifampin appears static for the first several hours in both media, but the bacteria end up growing to the level of the control by 24 h and 48 h, respectively. Fosfomycin kills the E. coli for the first several hours, but by the end of the experiments, the bacterial densities are similar to that of the antibiotic free control. As we obtain qualitatively similar results in both the LB and highly buffered minimal medium, we find it unlikely that changes in pH would account for the results shown in LB. Also, as we see bacterial density suppression at 48 h, the drugs are likely not decaying to a sufficient degree to allow for the bacteria to overcome the drug effects.

## DISCUSSION

The diagnosis of the most frequent diseases and protocols for their treatment are commonly based on relatively few estimated parameters like cholesterol levels, blood pressure, white cell counts, and glucose concentrations. In the case of antibiotic treatment, this parametric reductionism is in the application of the minimum concentration of the antibiotic required to prevent the replication of the bacteria presumed to be responsible for the infection, the MIC. As the unique measure of the pharmacodynamics, MICs are employed as both a measure of the susceptibility of the bacteria to the drug and for the design of dosing regimens for treatment of infections with those bacteria (8, 20). Under *in vivo* conditions, the local bacterial concentration at the site of infection, the replication rate, the available nutrients, the antibiotic concentration, and the effect of immune response are highly heterogeneous in time and space. In addition, under *in vivo* conditions within tissues, most bacteria are likely exposed to sub-MICs of drugs derived to an antibiotic gradient, and not to the drug concentration present in the serum (21). A single, simple test will be insufficient to cover these diverse and changing conditions. Efforts to design culture media mimicking the tissue environment, referred to by Ersoy et al. as the “fundamental flaw in the paradigm for antimicrobial susceptibility testing” (22), could slightly improve the *in vitro/in vivo* correlation, but these medias cannot evade the inconvenience of environmental heterogeneity (23–28). Our approach is evaluating the effects of nutrients and limiting resources on MIC. Glucose is likely not the main carbon source for E. coli in the host (29), but limitations *in vitro* of glucose or other carbon sources mimic the effect of shortage of other possible nutrients. The results of this study with eight antibiotics and E. coli illustrate the limitations of MICs for these purposes and also those of more comprehensive measures of the PD of antibiotics, such as Hill functions (30).

MICs are officially estimated with low densities of bacteria introduced into rich media, most common broths such as LB or MH (14, 31). Our results demonstrate that estimates of this parameter for azithromycin, tetracycline, and gentamicin obtained in highly buffered glucose-limited minimal medium are markedly lower than those estimated in broth. We have also shown that carbon source plays a large role in the determination of the MIC, particularly for azithromycin and fosfomycin. This is an indication that both nutrient limitation and choice of nutrient plays a large role in the determination of MIC (17, 32). It is to be noted that nutrient limitation and antibiotic action might both influence the bacterial stringent response, where (p)ppGpp “alarmone” levels lead to slower growth of the bacteria and effectively generate persister cells (33, 34). These population heterogeneities, reflected in our work, reveal a major limitation in the method for estimating MIC. Most notably, this singular pharmacodynamic parameter inherently cannot account for the possibility of rapid emergence of antibiotic resistance.

In addition to MICs, pharmacodynamic Hill functions provide information about the relationship between the concentration of the antibiotic and the dynamics and rates of growth of bacteria exposed to the drug (30). The parameters of Hill functions are estimated from the antibiotic concentration-dependent growth rates of the bacteria with the assumption being that these drugs only act by modifying these rates and do not take into account changes in any other bacterial growth parameters (30). The results of this and previous studies (35) question the validity of this assumption.

Our original model, a modification of the Hill functions which allows for resource consumption, predicts that if at sub-MICs all antibiotics did was to reduce the growth rate of the exposed bacteria, their populations would achieve the same stationary-phase densities but do so at times that increase with the concentration of the antibiotic. To an extent that varies among drugs, sub-MICs of antibiotics increase the time before the bacteria start to grow, the lag. This effect is most prominent among drugs that alter cellular metabolism. Antibiotics slowing metabolic processes likely slow development of cellular structures, as all structural elements of the cell will derive from glucose metabolism, which would be seen as an increase in the lag time. Also unanticipated by our model extending the Hill functions is that the maximum density to which the bacteria grow is dependent on the antibiotic concentration. Though, our updated model, which varies the conversion efficiency of glucose with an increasing concentration of an antibiotic, can account for these effects.

One possible explanation for why sub-MICs of antibiotics lower the densities to which the exposed populations of bacteria grow is that these drugs reduce the efficacy of use of the limiting resource. Stated another way, the bacteria exposed to subinhibitory antibiotic concentrations require more resource for replication. The effect of antibiotics on bacterial nutritional efficiency can explain this result. Certainly, antibiotic exposure decreases the metabolic flux, in that there is a waste of intracellular metabolites that are synthesized but not integrated into metabolic pathways. For instance, exposure to chloramphenicol and rifampin results in an accumulation of amino acids, nucleotides, and lipids (36). Similarly, beta-lactam exposure induces a futile cycle of cell wall synthesis and degradation, which depletes cellular resources (37). There is also emerging evidence that sub-MICs of antibiotics can influence nonessential tRNA and RNA modifications, leading to unnecessary energy expenditures (38). Such energetic waste should be compensated by the consumption of nutrients that do not necessarily contribute to the net growth rate.

Consistent with this hypothesis at sub-MICs, all of the antibiotic treated cultures fail to grow up to the glucose-limited density of the controls, but yet all of the glucose is utilized in that no or very little free glucose is detectable, while at super-MICs the bacterial densities are suppressed, and a substantial amount of free glucose remains. As a departure from this trend, the cultures treated with super-MICs of ceftriaxone use more free glucose than the cultures treated with super-MICs of the other drugs, but yet a large amount of glucose is still present. It has not escaped our notice that based on Fig. 8, this beta-lactam over the first 8 h appears very similar to the bacteriostatic drugs. Could it be that in these first few hours, the growth rate is equal to the death rate (39) so that these bacteria are still utilizing the free glucose? Alternatively, ceftriaxone is known to produce filaments due to PBP3 inhibition, could it be that these multicellular filaments have an increased surface/volume ratio (favoring glucose uptake) and perhaps an increased metabolic requirement when compared to their ancestor cells?

We are confident that the results of the experiments performed here are broadly replicable across antibiotic drug classes and media type (rich versus minimal) but recognize there are caveats to consider. First, that our study was performed with just one laboratory strain of E. coli, MG1655. Second, the study was also conducted with only two media. One of which was LB which we used to represent nutrient rich conditions, though MICs for clinical purposes are officially performed in MH. We postulate our choice of strain and media will not change the observed phenomena qualitatively. That said, we do anticipate changing strains or media will quantitatively change the results observed here. More specifically, we expect the MICs obtained will depend on both the bacterial strain and media.

Advancement in the way we measure and understand pharmacodynamics should have therapeutic implications. Our main difficulty is the heterogeneity of the microecological conditions at the site of infection (such as immunological factors, nutritional deficiencies, local pH or oxygen availability, access of the drugs, or protein binding). Factors which are difficult or impossible to reproduce under *in vitro* conditions. In laboratory tests, the most used PD parameter, the MIC, certainly allows for the comparison of antibiotic susceptibility between two isolates of the same species under identical *in vitro* conditions. However, MIC is an insufficient PD marker for several reasons. First, the antibiotic effect at subinhibitory concentrations is not considered. Second, this parameter does not tell us anything about the susceptibility of a given organism at different cell densities. Third, it tells us nothing about changes in growth parameters such as the effect on lag, average growth rate, final density, or overall rate of drug-mediated killing. Lastly, MIC indicates nothing about postantibiotic effects, resistance, persistence, or heteroresistance. All these pharmacodynamic dimensions change the effect of antibiotics *in vivo* as the concentration of the drug changes over time, as predicted by the PK. Without understanding these effects, the operative linkage of PK/PD is insufficient to obtain robust predictions about antibiotic efficacy. When MIC is used as the sole PD parameter, this insufficiency is even more apparent. Most PK/PD approaches look at one of three PK parameters: either maximum concentration of the antibiotic to MIC, drug exposure to MIC (which is to say area under the curve to MIC), or the time that the drug concentration is above the MIC (40). Most importantly, the development of novel antibiotics should consider these PD complexities and adopt a nonreductive approach which is not simply limited to MIC. An antibiotic should be more effective than another as shown by a holistic superiority-test, considering multiple parameters for PD. These tests should take into account: the effect of the density of the starting bacterial population, the effectiveness of the drug on bacteria in different stages of growth, the ability to reduce bacterial growth rate, increase lag time, and decrease final population size, as well as decrease the number of persisters. Optimally, all these advantages would also reduce the length of treatment, the emergence of antibiotic resistance, and the recurrent infections, as well as the possibility of transmission of pathogens to other hosts. However, the usual microbiological PD tests in drug development do not include these calculations, benefits which would go unrecognized by using an only-MIC-focused approach.

## MATERIALS AND METHODS

### Numerical solutions (simulations).

For our numerical analysis of the equations presented (equations 1–6), we used Berkeley Madonna, using parameters in the ranges estimated for E. coli. Copies of the Berkeley Madonna program used for these simulations are available at www.eclf.net.

### Growth media.

LB broth (244620) was obtained from BD. The DM (Davis Minimal) minimal base without dextrose (15758-500G-F) was obtained from Sigma-Aldrich (7 g/L dipotassium phosphate, 2 g/L monopotassium phosphate, 0.5 g/L sodium citrate, 0.1 g/L magnesium sulfate, 1 g/L ammonium sulfate). MH broth (M391-500g) was obtained from HiMedia. Glucose (41095-5000) was obtained from Acros, Succinic Acid (S-2378) was obtained from Sigma-Aldrich, Lactose (L3750) was obtained from Sigma-Aldrich, Maltose (M2250) was obtained from Sigma-Aldrich, Glycerol (G7757) was obtained from Honeywell and Fructose (161355000) was obtained from ACROS Organics. LB agar (244510) for plates was obtained from BD.

### Bacterial strains.

E. coli MG1655 was obtained from the Levin Lab Bacterial collection. This Whole Genome Shotgun project has been deposited at DDBJ/ENA/GenBank under the accession JAQIDJ000000000. The version described in this paper is version JAQIDJ010000000.

### Antibiotics.

Ciprofloxacin (A4556) was obtained from AppliChem Panreac, Tetracycline (T17000) was obtained from Research Products International, Fosfomycin (P5396) was obtained from Sigma-Aldrich, Chloramphenicol (23660) was obtained from USB, Rifampin (BP2679-1) was obtained from Fisher BioReagents, Ceftriaxone (C5793) was obtained from Sigma-Aldrich, Azithromycin (3771) was obtained from TOCRIS and Gentamicin (BP918-1) was obtained from Fisher BioReagents.

### Sampling bacterial densities.

The densities of bacteria were estimated by serial dilution in 0.85% saline and plating. The total density of bacteria was estimated on LB plates with 1.6% agar.

### Growth rate estimation.

Exponential growth rates were estimated from changes in optical density (OD600) in a Bioscreen C. For this, 24-h stationary-phase cultures were diluted in LB or glucose-limited liquid media to an initial density of approximately 10^5^ cells per mL. Five replicas were made for each estimate by adding 300 μL of the suspensions to the wells of the Bioscreen plates. The plates were incubated at 37°C and shaken continuously. Estimates of the OD (600 nm) were made every 5 min for 24 h in LB and 48 h in glucose-limited medium. Normalization, replicate means and error, growth rate, lag and maximum OD were found using a novel R Bioscreen C analysis tool accessible at https://josheclf.shinyapps.io/bioscreen_app.

### Sequencing and analyses.

Bacterial DNA was extracted using Promega’s (WI, USA) Wizard Genomics DNA purification kit (Cat# A1120) using the manufacturer’s protocol. The extracted DNA was quantified on Thermo Fisher’s NanoDrop OneC microvolume spectrophotometer (Cat# ND-ONEW). Samples were sent to the Microbial Genome Sequencing Center in Pittsburgh, PA, USA, for whole-genome sequencing on the Illumina NextSeq 2000 platform. Analysis of FASTAq files received from the Microbial Genome Sequencing Center were analyzed using Geneious Prime version 2022.0.1.

### MICs.

Antibiotic MICs were estimated using a 2-fold microdilution procedure as described in Jorgensen et al. (41).

### Antibiotic time-kills.

For these experiments, overnight cultures of E. coli MG1655 were added to LB broth or DM 1,000 μg/mL glucose liquid media at an initial density of approximately 10^5^ cells per mL, followed by 1 h of incubation at 37°C and shaken continuously. After incubation, antibiotics were added at the concentrations indicated in Fig. 4 and 5 and the initial densities of the cultures were estimated. The cultures with the antibiotics were incubated for 24 h in LB media and 48 h in DM glucose and estimates of the viable cell densities were made at 15 min, 30 min, 45 min, 1 h, 1.5 h, 2 h, 3 h, 4 h, and 24 h for LB cultures and 1 h, 2 h, 3 h, 4 h, 5 h, 6 h, 7 h, 8 h, 24 h, and 48 h for the DM glucose liquid media cultures.

### Glucose assay.

Glucose concentration was measured by the dinitrosalicylic colorimetric method as described in Miller (42).

### Data availability.

All the data generated are available in this manuscript and its supporting supplemental material.
