# Supplementary material for: What’s the Matter with MICs: Bacterial Nutrition, Limiting Resources, and Antibiotic Pharmacodynamics
Source: Microbiol Spectr. 2023 May 3;11(3):e04091-22. doi: 10.1128/spectrum.04091-22 (PMC10269441; doi:10.1128/spectrum.04091-22)
Supplement: Supplemental file 1 — Supplemental material. Download spectrum.04091-22-s0001.pdf, PDF file, 2.9 MB [file spectrum.04091-22-s0001.pdf]

Supplementary Information for

What's the matter with MICs: bacterial nutrition, limiting resources, and antibiotic pharmacodynamics

Brandon A. Berryhill, Teresa Gil-Gil, Joshua A. Manuel, Andrew P. Smith, Ellie Margollis, Fernando Baquero, Bruce R. Levin*

*Corresponding Author
Email: blevin@emory.edu

This file includes:

Figures S1 to S10
Tables S1

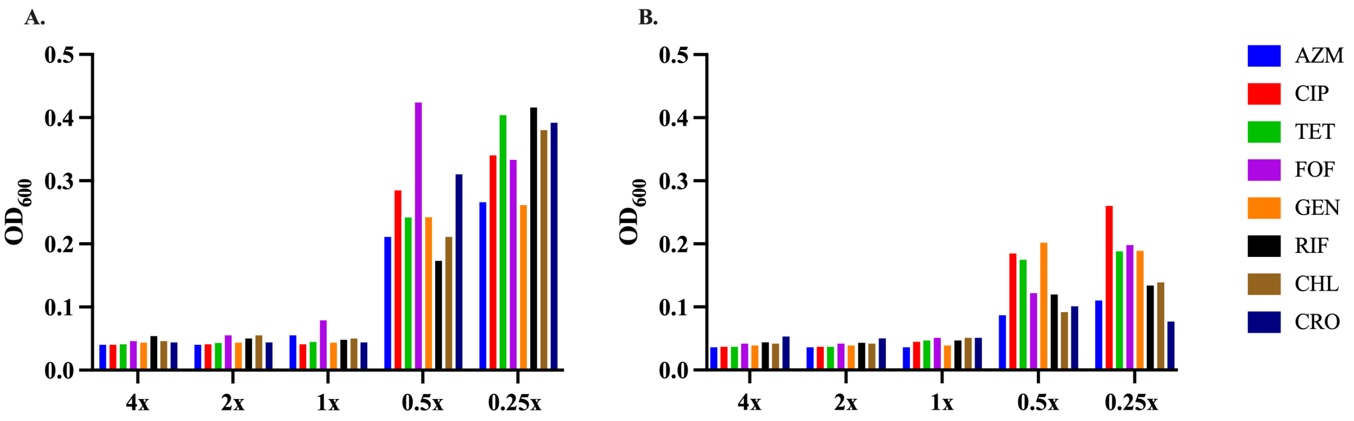

Fig. S1. MIC ooze. Antibiotic MICs were performed using a 2-fold microdilution procedure and after 24 hours in **(A)** LB and 48 h in **(B)** glucose-limited minimal medium the final optical density (OD600nm) was measured at different concentrations of antibiotic.

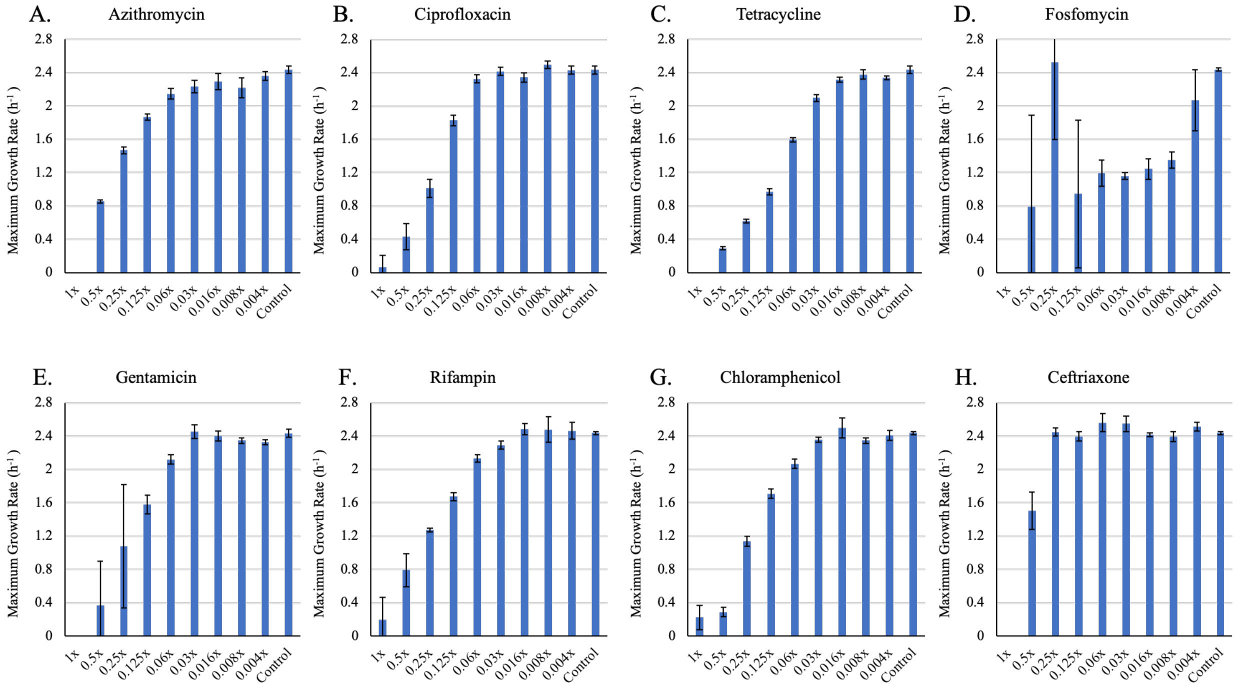

Fig. S2. Changes in maximum growth rate (v_{MAX}) of E. *coli* MG1655 exposed to different sub-MIC concentrations of eight antibiotics for 24 hours in LB. Bars are representative of the average of five technical replicas. Each concentration is shown as a fraction of the MIC for the noted drug (**A**) Azithromycin (**B)** Ciprofloxacin (**C)** Tetracycline (**D)** Fosfomycin (**E)** Gentamicin (**F)** Rifampin (**G)** Chloramphenicol (**H)** Ceftriaxone.

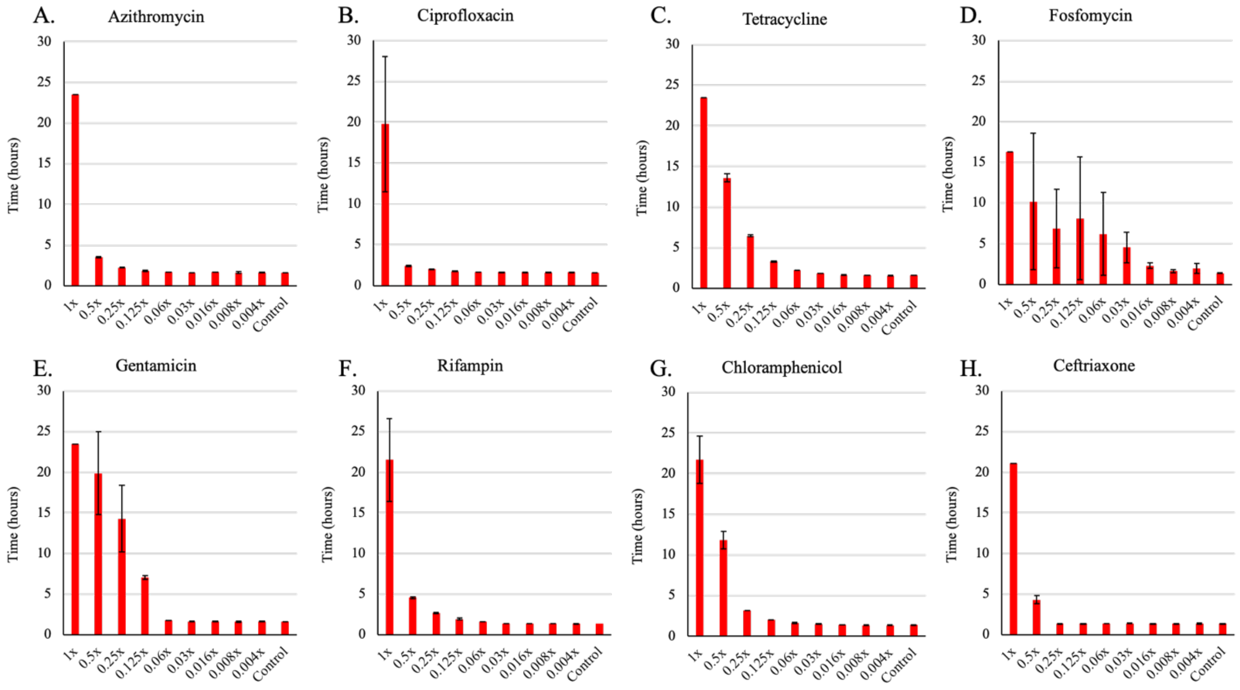

Fig. S3. Changes in the time before the bacteria start to grow (lag) of E. *coli* MG1655 exposed to different sub-MIC concentrations of eight antibiotics for 24 hours in LB. Bars are representative of the average of five technical replicas. Each concentration is shown as a fraction of the MIC for the noted drug **(A)** Azithromycin **(B)** Ciprofloxacin **(C)** Tetracycline **(D)** Fosfomycin **(E)** Gentamicin **(F)** Rifampin **(G)** Chloramphenicol **(H)** Ceftriaxone.

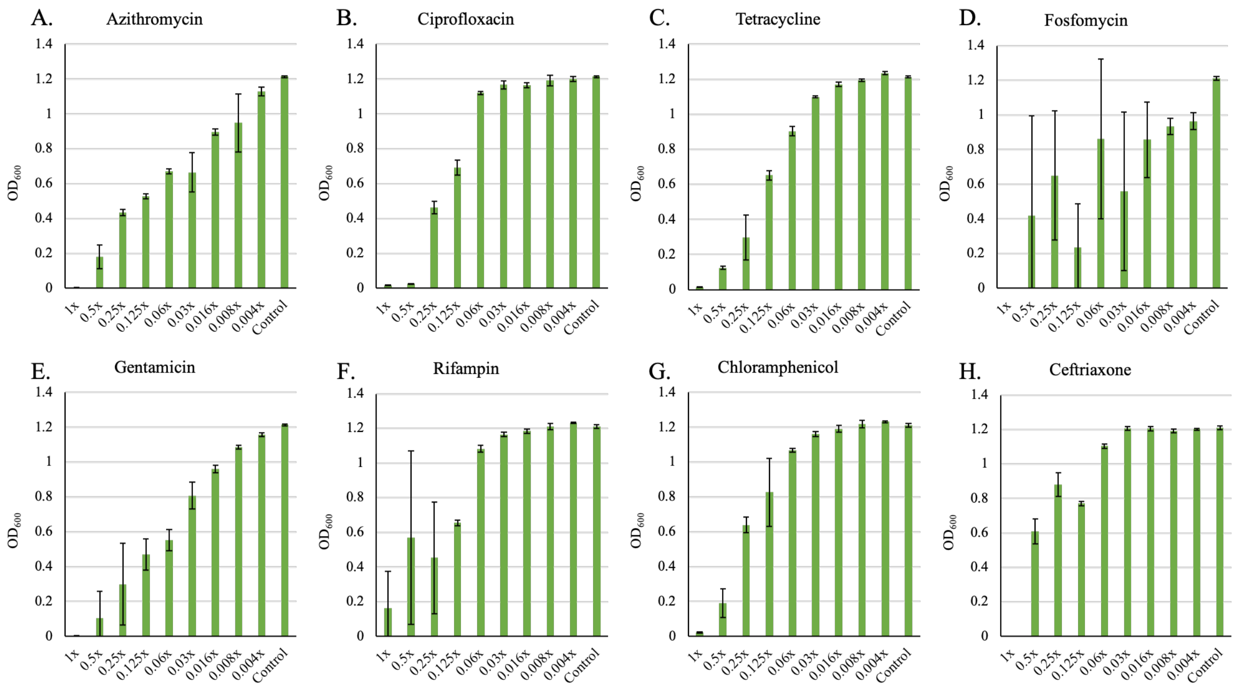

Fig. S4. Changes in the maximum optical density (OD 600nm) of E. *coli* MG1655 exposed to different sub-MIC concentrations of eight antibiotics for 24 hours in LB. Bars are representative of the average of five technical replicas. Each concentration is shown as a fraction of the MIC for the noted drug (**A**) Azithromycin (**B**) Ciprofloxacin (**C**) Tetracycline (**D**) Fosfomycin (**E**) Gentamicin (**F**) Rifampin (**G**) Chloramphenicol (**H**) Ceftriaxone.

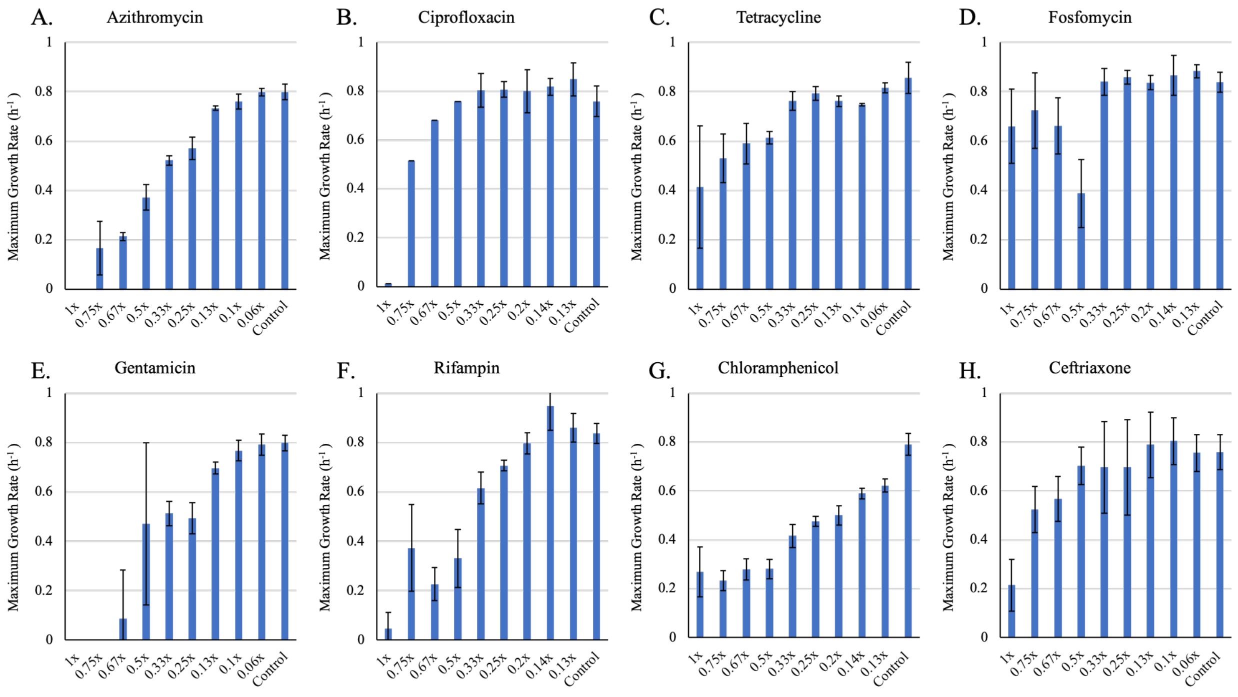

Fig. S5. Changes in maximum growth rate (v_{MAX}) of E. *coli* MG1655 exposed to different sub-MIC concentrations of eight antibiotics for 48 hours in glucose-limited minimal medium. Bars are representative of the average of five technical replicas. Each concentration is shown as a fraction of the MIC for the noted drug (**A)** Azithromycin (**B)** Ciprofloxacin (**C)** Tetracycline (**D)** Fosfomycin (**E)** Gentamicin (**F)** Rifampin (**G)** Chloramphenicol (**H)** Ceftriaxone.

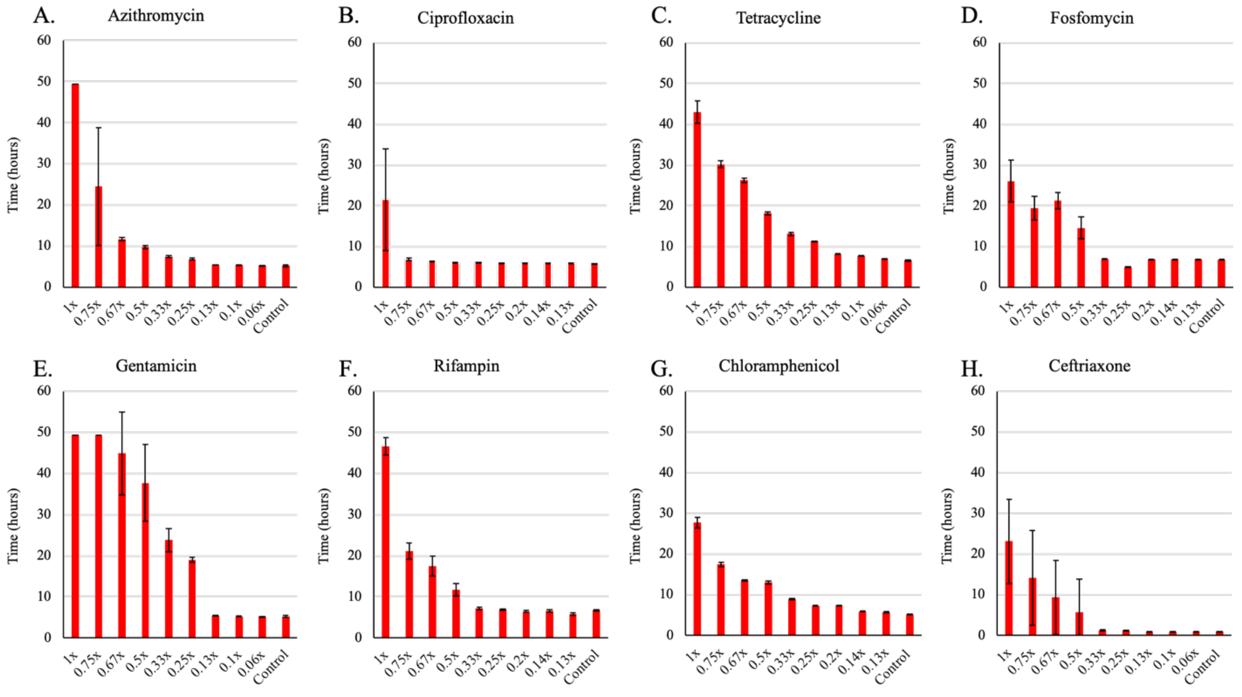

Fig. S6. Changes in the time before the bacteria start to grow (lag) of E. *coli* MG1655 exposed to different sub-MIC concentrations of eight antibiotics for 48 hours in glucose-limited minimal medium. Bars are representative of the average of five technical replicas. Each concentration is shown as a fraction of the MIC for the noted drug **(A)** Azithromycin **(B)** Ciprofloxacin **(C)** Tetracycline **(D)** Fosfomycin **(E)** Gentamicin **(F)** Rifampin **(G)** Chloramphenicol **(H)** Ceftriaxone.

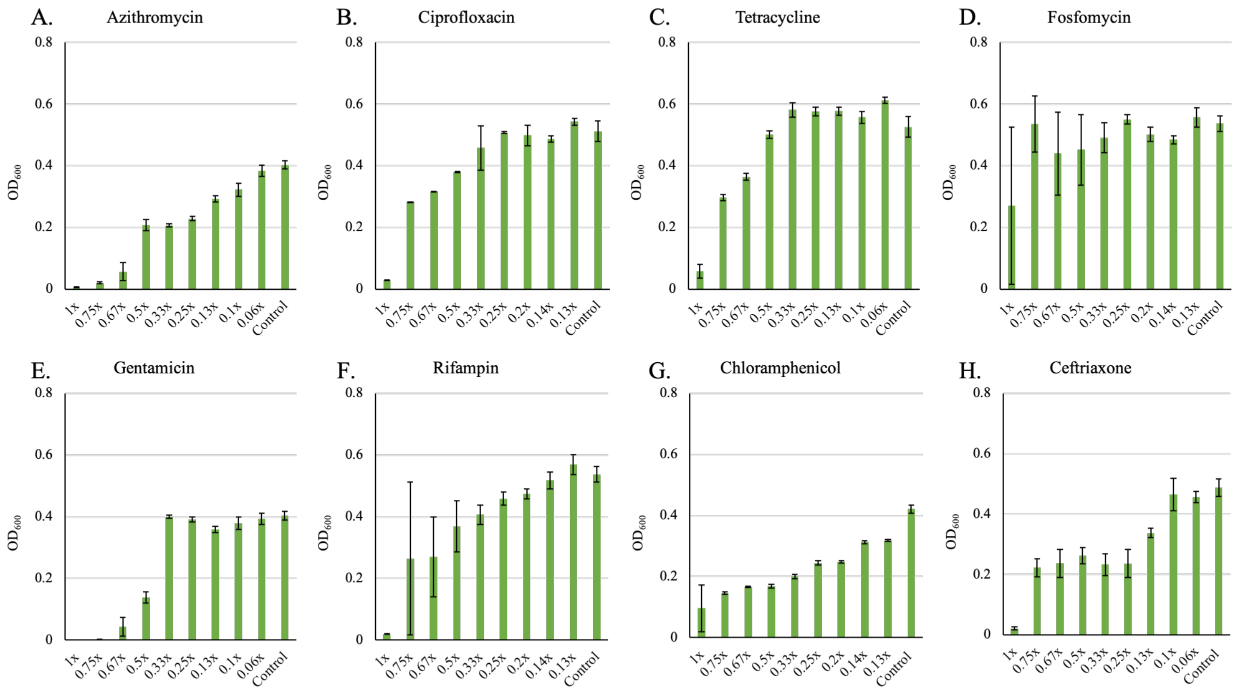

Fig. S7. Changes in the maximum optical density (OD 600nm) of E. *coli* MG1655 exposed to different sub-MIC concentrations of eight antibiotics for 48 hours in glucose-limited minimal medium. Bars are representative of the average of five technical replicas. Each concentration is shown as a fraction of the MIC for the noted drug (**A**) Azithromycin (**B)** Ciprofloxacin (**C)** Tetracycline (**D)** Fosfomycin (**E)** Gentamicin (**F)** Rifampin (**G)** Chloramphenicol (**H)** Ceftriaxone.

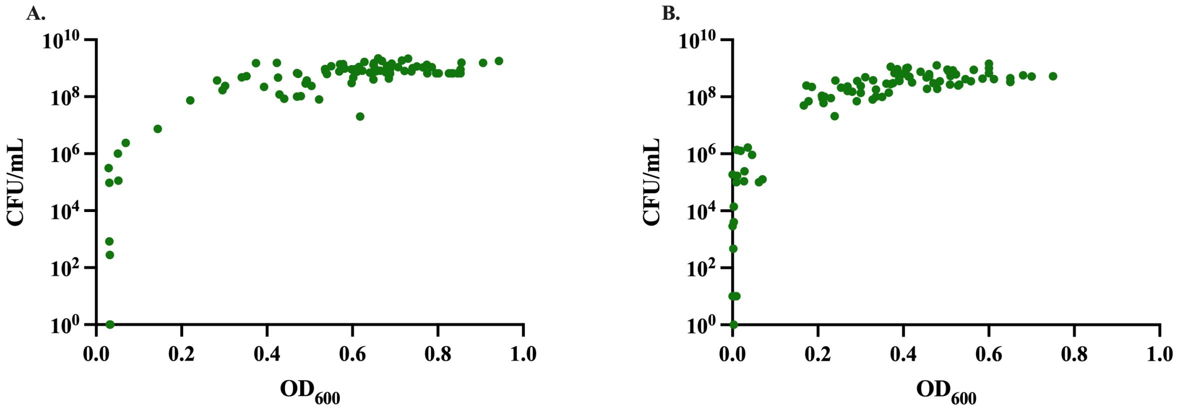

Fig. S8. Relation between optical density and viable colony forming units (CFU) of E. *coli* MG1655 cultures exposed to sub- and super- MIC concentrations of the eight antibiotics in **(A)** LB after 24 hours and in **(B)** glucose-limited medium after 48 hours of incubation.

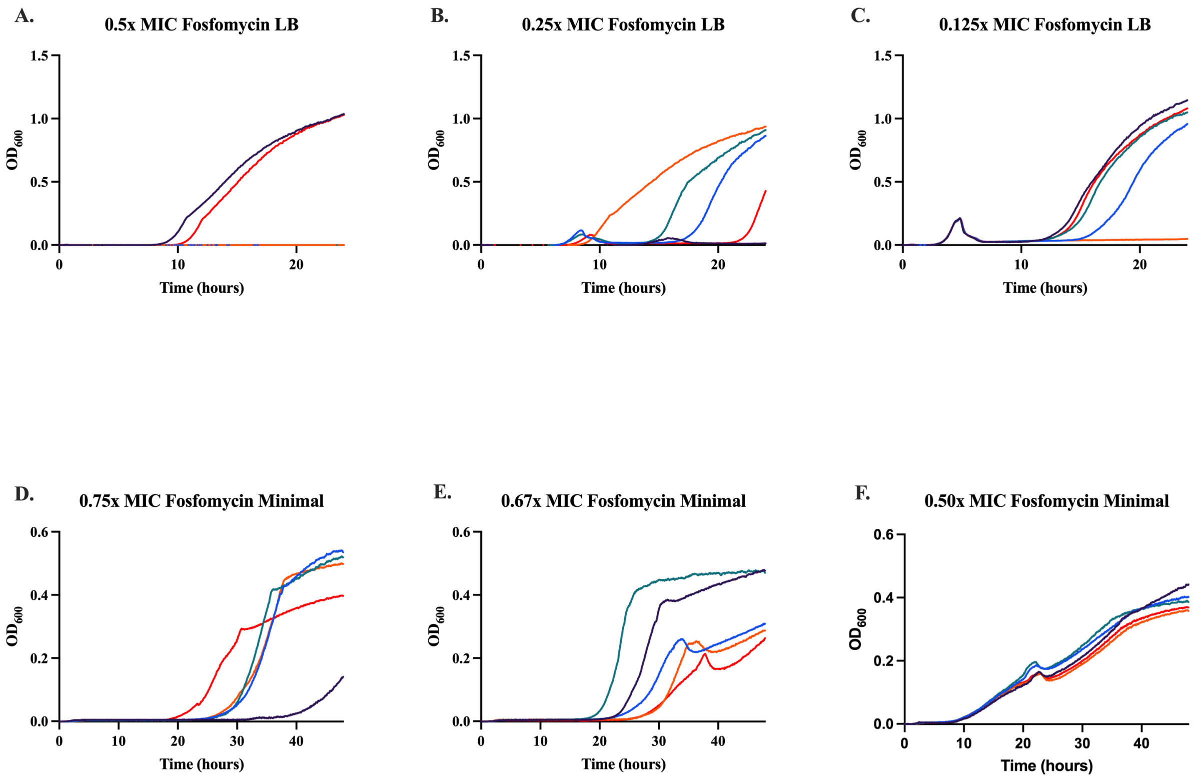

Figure S9. Growth dynamics in LB and glucose-limited medium at different fosfomycin concentrations. Changes in optical density (600nm) of *E. coli* MG1655 exposed to different fosfomycin concentrations for 24 hours in LB and 48 h in glucose-limited medium. Lines are representative of one technical replica and normalized to the time zero optical density. Individual replicate curves show how resistance came up at different fosfomycin concentrations.

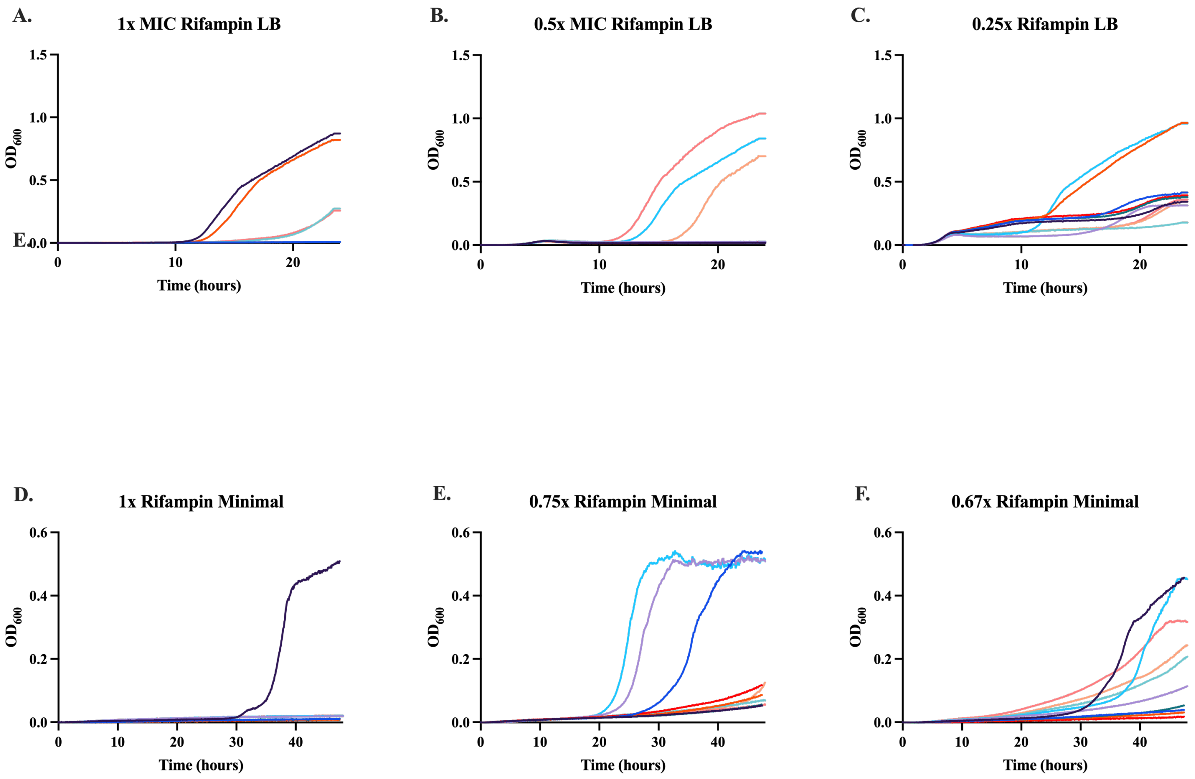

Figure S10. Growth dynamics in LB and glucose-limited medium at different rifampin concentrations. Changes in optical density (600nm) of *E. coli* MG1655 exposed to different rifampin concentrations for 24 hours in LB and 48 h in glucose-limited medium. Lines are representative of one technical replica and normalized to the time zero optical density. Individual replicate curves show how resistance came up at different rifampin concentrations.

Table S1. Estimated MICs of *E. coli* MG1655 for eight antibiotics in limited minimal medium with different carbon sources

	MIC ($\mu g/mL$)					
Antibiotic	Glucose	Succinate	Lactose	Maltose	Glycerol	Fructose
AZM	6.25	1.56	3.13	3.13	1.56	3.13
CIP	0.03	0.03	0.016	0.016	0.016	0.016
TET	0.8	0.8	0.8	0.8	0.8	0.8
FOF	25	3.125	12.5	12.5	25	12.5
GEN	0.75	0.37	0.75	0.37	0.37	0.37
RIF	12.5	12.5	12.5	12.5	12.5	12.5
CHL	6.25	6.25	6.25	6.25	3.13	6.25
CRO	0.03	0.006	0.03	0.012	0.03	0.012
